# Osmoregulation and the Hypothalamic Supraoptic Nucleus: From Genes to Functions

**DOI:** 10.3389/fphys.2022.887779

**Published:** 2022-05-24

**Authors:** André Souza Mecawi, Wamberto Antonio Varanda, Melina Pires da Silva

**Affiliations:** ^1^ Laboratory of Molecular Neuroendocrinology, Department of Biophysics, Paulista School of Medicine, Federal University of São Paulo, São Paulo, Brazil; ^2^ Department of Physiology, Faculty of Medicine of Ribeirão Preto, University of São Paulo, Ribeirão Preto, Brazil; ^3^ Laboratory of Cellular Neuroendocrinology, Department of Biophysics, Paulista School of Medicine, Federal University of São Paulo, São Paulo, Brazil

**Keywords:** magnocellular neurosecretory cells, osmoregulation, gene plasticity, ions channels, transporters, supraoptic nucleus

## Abstract

Due to the relatively high permeability to water of the plasma membrane, water tends to equilibrate its chemical potential gradient between the intra and extracellular compartments. Because of this, changes in osmolality of the extracellular fluid are accompanied by changes in the cell volume. Therefore, osmoregulatory mechanisms have evolved to keep the tonicity of the extracellular compartment within strict limits. This review focuses on the following aspects of osmoregulation: 1) the general problems in adjusting the “milieu interieur” to challenges imposed by water imbalance, with emphasis on conceptual aspects of osmosis and cell volume regulation; 2) osmosensation and the hypothalamic supraoptic nucleus (SON), starting with analysis of the electrophysiological responses of the magnocellular neurosecretory cells (MNCs) involved in the osmoreception phenomenon; 3) transcriptomic plasticity of SON during sustained hyperosmolality, to pinpoint the genes coding membrane channels and transporters already shown to participate in the osmosensation and new candidates that may have their role further investigated in this process, with emphasis on those expressed in the MNCs, discussing the relationships of hydration state, gene expression, and MNCs electrical activity; and 4) somatodendritic release of neuropeptides in relation to osmoregulation. Finally, we expect that by stressing the relationship between gene expression and the electrical activity of MNCs, studies about the newly discovered plastic-regulated genes that code channels and transporters in the SON may emerge.

## Introduction

The water molecule has very particular physicochemical properties, mainly due to an asymmetrical distribution of charges. Even though the molecule is neutral, it behaves like a structural dipole, allowing interaction through hydrogen bonding and giving rise to a property known as cohesion. Also, by being a dipole, it can interact with ions of both polarities, entering the crystal structure and dissolving the salt. This property makes water a universal solvent, at least in terms of biological relevance in maintaining the process of life. Therefore, variations in the water activity inside and/or outside a cell will be accompanied by changes in the activity of electrolytes, in general, and in the structure of macromolecules in particular, two fundamental properties necessary for biochemical reactions to occur appropriately.

In higher organisms, the total body water accounts for 60–70% of weight and is distributed among several defined compartments. In the 19th century, Claude Bernard (Gross, 1998) recognized that cells live in the so-called “milieu interieur”, which is aqueous in nature, whose composition and volume must be within strict limits for life to be maintained. The existence of water was a prerequisite for the establishment of life on earth, serving not only as a polar medium for dissolving hydrophilic substances but also acting in the organization of apolar and amphipathic molecules such as the structuring of cell membranes (Tanford, 1978; Krimm, 1980). Since life probably began in a pristine ocean with high Na^+^ concentration, mammals inherited from primitive multicellular ancestors an aqueous extracellular compartment having ionic concentrations corresponding to approximately 1/3 of those observed in the ocean, with Na^+^ as the primary ion in the extracellular medium.

The long-timescale required by the evolutionary processes that resulted in today’s highly complex organisms is very intricate and essentially dependent on the emergence of specialized tissues to control and maintain the “milieu interieur” in a steady-state condition. That is especially true concerning the maintenance of body water content and osmolality. The conquest of dry environments by vertebrates was especially challenging from the standpoint of keeping an osmotic equilibrium between the intra- and extracellular compartments. Undoubtedly, physiological systems' emergence and efficiency gain specialized in 1) sensing osmotic changes; 2) coordinating adequate multimodal defense responses; and 3) effective management of electrolytes and water fluxes across the plasma membrane. These were essential for attaining the great evolutionary success of terrestrial vertebrates.

In mammals, the hypothalamic-neurohypophysial system (HNS) is the crucial neuroendocrine apparatus responsible for controlling extracellular osmolality. The HNS fulfills the three central aspects involved in osmoregulation mentioned above: 1) it is composed of specialized osmosensing magnocellular neurosecretory cells (MNCs), located in the hypothalamic supraoptic and paraventricular nuclei (SON and PVN); 2) it coordinates the multimodal responses by secreting the neuropeptides arginine-vasopressin (AVP) and oxytocin (OXT) into the circulation; and 3) it finely adjusts kidney function, the primary organ involved in the control of extracellular volume and composition. The present review focuses on the basic concepts related to water and electrolyte movement across the plasma membrane and discusses the molecular and cellular mechanisms involved in osmosensing by the MNCs in mammals. We give special attention to the processes at the plasma membrane involving channels and transporters, which regulate cellular excitability and the osmosensory processes in MNCs. Based on the recently described changes in the supraoptic nucleus transcriptome in response to prolonged dehydration, we also discuss the molecular plasticity of the NHS and its consequences for osmoreception. Finally, we pay attention to somatodendritic release of neuropeptides in relation to osmoregulation.

### Water Movement Across the Plasma Membrane

Despite the high intensity of water exchange between the extra- and intracellular compartments, the process is essentially passive: water always moves according to its chemical gradient. In other words, all one needs to induce a net water flux across cell membranes is an osmotic (Δπ) and/or a hydrostatic pressure gradient (ΔP). This fact has an interesting experimental implication: similar results should be obtained by applying either an osmotic or a hydrostatic pressure gradient.

Another point of interest is that cell membranes, in general, show considerable osmotic water permeability. Small values are found in cells of the thick ascending limb of Henle’s loop in mammalian kidneys and the highest are found in the plasma membrane of red blood cells (Reuss, 2008). This property is a function of both the proteins present in the membrane and those in the phospholipid bilayer itself. Bilayers containing sphingolipids and/or cholesterol have lower water permeability compared to bilayers formed exclusively of phospholipids (Finkelstein and Cass, 1967; Finkelstein, 1976). The asymmetrical distribution of lipids between the bilayer leaflets and the sequestration of specific lipids in rafts also influence the permeability to water (Hill and Zeidel, 2000; Rawicz et al., 2008; Delpire and Gagnon, 2018). The water permeability is also dependent on the presence of water channels of the aquaporin family (Zeidel et al., 1992; Su et al., 2020). These channels have a ubiquitous distribution. While AQP2 (among others) is present in the collecting ducts of the kidney and their expression is mainly controlled by AVP (Fushimi et al., 1993), AQP1 and AQP4 have been described in astrocytes and ependymocytes in the brain (Verkman et al., 2011, 2017). In any case, water has an equilibrium distribution across the plasma membrane. An imbalance in the osmotic and/or a hydrostatic pressure gradient will result in a net influx or outflux of water, impacting the cell volume.

Interestingly, there is no single molecule responsible for sensing osmotic changes in the cell. Instead, changes in osmolality result in changes in the water activity. Therefore, the ionic strength of the intracellular solution can vary with the cell volume, leading to dysfunctional biochemical processes, impairment of electrical excitability, protein phase separation, changes in membrane tension, etc. (Model et al., 2021).

### Cell Volume

When exposed to challenges in osmolality, cells first respond as osmometers, and then the influx or efflux of solutes is modified to counterbalance volume changes due to the concomitant water flow. This biphasic response involves the activation of a repertoire of membrane channels and transporters to circumvent the volume changes.

In response to a decrease in extracellular osmolality, the volume of the cell first increases in proportion to the imposed reduction of osmolality. Then an outflow of volume primarily induced by a net flux of osmotically active solutes directed from the intra-to the extracellular space is observed. This response is known as regulatory volume decrease (RVD) and has a time course dependent on the cell type (Grinstein et al., 1982; Hazama and Okada, 1988; Sato et al., 2011; Okada et al., 2019). For more details about cell volume regulation, see Strange, 1992, Strange, 2004). Therefore, several mechanisms have evolved to maintain the cell volume under isotonic conditions and restore it when changes in osmolality are imposed on the system. The ubiquitous Na^+^/K^+^ ATPase was one of the first mechanisms to be recognized in this respect. The active movement of Na^+^ out of the intracellular compartment linked to the generally low membrane permeability to Na^+^ place this ion preferentially in the extracellular compartment, acting as an osmotically effective solute, as recognized long ago (Leaf, 1959; Tosteson and Hoffman, 1960; Ussing, 1960). To counterbalance volume changes, cells can lose K^+^ when a reduction of volume is necessary or gain Na^+^ to increase their volume. Because a movement of anions is coupled to the fluxes of Na^+^ and K^+^, a repertoire of membrane channels and transporters is used to cope with the osmotic challenges. HCO_3_^−^ and Cl^−^ are the main counterions participating in the process. In this way, the coupled exchange of Na^+^/H^+^ and Cl^−^/HCO_3_^−^ contributes to controlling the intracellular pH and cell volume recovery in response to a hyperosmotic challenge. The concerted action of these two types of transporters ultimately results in a net gain of NaCl and the associated volume of water by the cell (Grinstein and Rothstein, 1986; Humphreys et al., 1995; Hughes et al., 2010; Romero et al., 2013; Li et al., 2021).

Another important class of transporters is the CCC family (cation-chloride cotransporters), which couples the movement of Cl^−^, Na^+^, and K^+^ across the cell membrane to that of water, to counterbalance the effects of changes in tonicity. Among these cotransporters are NKCC1 and NKCC2, which import chloride, sodium, and potassium to the cell, and KCC2, which in turn exports K^+^ and Cl^−^ from the cytoplasm. Besides their role in setting the electrical excitability of cells, via control of the intracellular chloride concentration (Russell, 2000; Blaesse et al., 2009), they also participate in the response to hypotonicity: while KCC is activated by dephosphorylation of serine-threonine residues, NKCC1 is inhibited by this process (Bize et al., 1999), resulting in a net loss of KCl by the cell and reduction of its volume (Chew et al., 2019)

Those transporters act in synchrony with several ion channels, particularly chloride channels. Numerous putative anion channels are linked to volume regulation and are activated by hypotonicity, i.e., the net entrance of water to the cytoplasm. Cell swelling has been shown to activate large-conductance chloride channels in several vertebrate cell types (Grinstein et al., 1982; Doroshenko and Neher, 1992; Nilius et al., 1994; Jentsch, 2002; Wang et al., 2005; Poletto Chaves and Varanda, 2008; Okada et al., 2019). We should note that the activity of a volume regulated anion channel (VRAC) has been recently associated with a protein of the LRRC8 family, coded by several homologous genes of the mammalian genome (Bize et al., 1999; Qiu et al., 2014; Voss et al., 2014; Pedersen et al., 2016; König and Stauber, 2019).

### Magnocellular Neurosecretory Cells and Brain Osmoreception

Since the maintenance of cell volume is highly dependent on the osmolality of the extracellular fluid, systems have been selected for sensing and correcting changes in both volume and osmolality of the “milieu interieur”. Although most cells face constant osmotic challenges and may change their activity in response to such perturbations, only a small number of them participate in specific mechanisms aimed to restore body fluid homeostasis. In a series of pioneering experiments, Verney and collaborators “...postulated that the neurohypophysis was functionally linked with sensory elements sensitive to changes in the osmotic pressure of their vascular environment. Such elements were termed osmoreceptors...” (see Jewell and Verney, 1957). Based on the latest advances in the field, it is clear that the osmoreceptors are not formed by a single molecular entity. Instead, each osmoreceptor unit must be associated with a cellular entity able to couple changes in osmolality, resultant from changes in the activity of water, to changes in its plasma membrane electrical activity. So far, the commonly accepted evidence indicates that the brain osmoreceptors are comprised by a very small number of specialized neurons able to transduce changes in their cell volume into electrophysiological responses (Prager-Khoutorsky et al., 2014). Several pioneer studies have identified two types of osmoreceptor neurons: 1) a subset of neurons located in the *lamina terminalis*, at the *organum vasculosum lamina terminalis* (OVLT) and the subfornical organ (SFO), two circumventricular structures outside the blood-brain-barrier; and 2) the neurosecretory MNCs, located in the PVN and SON (for a review, see Bourque, 2008). These two subsets of neurons (circumventricular and neurosecretory) are functionally connected, i.e., OVLT and SFO neurons send afferents to the SON and PVN to control their electrical activity, and consequently the neurosecretory response (Johnson and Gross, 1993; Bourque and Oliet, 2003). While cells in the OVLT and SFO are mainly involved in conveying thirst and/or sodium appetite information to the cortex (Egan et al., 2003; Bourque, 2008; Allen et al., 2017; Matsuda et al., 2017). The MNCs of both SON and PVN produce and secrete AVP and OXT through the neurohypophysis to the circulation. Those osmosensitive neurons respond to changes as low as 1% in osmolality in an independent manner by modifying the frequency and/or the discharge pattern of action potentials (Poulain and Wakerley, 1982).

In this context, the MNCs play unique roles in the osmoregulatory process because they have the following basic properties: 1) intrinsic osmosensitivity; 2) integration of osmoreceptive information from the circumventricular organs; and 3) synthesis and secretion of two of the main hormones controlling renal water and sodium excretion. While the SON has a very homogenous neuronal population (the vast majority are MNCs), send axonal projections mainly to the neurohypophysis, and produces mainly AVP and OXT, the PVN is more complex. Besides the AVP and OXT MNCs projecting to the neurohypophysis, the PVN also has several subsets of parvocellular neurons which produce other neuropeptides (such as CRH, TRH, SST, etc.) and project to the median eminence to control the adenohypophysis function; to the brainstem to control autonomic outflow; and to limbic structures to control behavioral responses (de Souza Mecawi et al., 2015; Dos-Santos et al., 2019). Thus, here we focus on the osmosensory processes, electrophysiological responses, and the molecular plasticity of the MNCs of the SON and their related glial cells.

The SON is located bilaterally to the optic chiasm and integrates afferent information from other areas involved in the maintenance of the osmolality of the extracellular fluid (Yang et al., 1994; Richard and Bourque, 1995). As previously described, MNCs not only secrete AVP and OXT under the control of other osmosensory areas, but also respond intrinsically to direct osmotic stimulation. This fact has been demonstrated by results from experiments performed in intact anesthetized animals (Brimble and Dyball, 1977; Poulain et al., 1977; Wakerley et al., 1978; Poulain and Wakerley, 1982), in slices where the synaptic inputs have been pharmacologically blocked (da Silva et al., 2013, 2015), and in isolated MNCs from both SON and PVN (Oliet and Bourque, 1992, 1993; Zhang et al., 2007). It has been demonstrated that changing cell volume by osmotic stimulation or direct pressure application with a patch pipette activates mechanosensitive ion channels in these cells (Oliet and Bourque, 1993; Zhang and Bourque, 2003). The question that arises here is: Which ultrastructural cellular component could be responsible for coupling changes in volume to electric plasma membrane responses? Super-resolution images obtained by structured illumination microscopy have revealed that MNCs contain many actin filaments and microtubules in their somata, with exclusive organization compared with cells in other brain regions, such as the cortex, as well as with hippocampal and other hypothalamic neurons (Prager-Khoutorsky et al., 2014). Additionally, the density/array of actin filaments and microtubules are substantially changed in response to salt-loading (Barad et al., 2020; Hicks et al., 2020), reinforcing the hypothesis that these elements participate in the mechanotransduction process. Based on the assumption that cytoskeleton components can redistribute forces across the lipid bilayer (Ingber, 1997), Zhang et al. (2007) proposed that osmosensory transduction depends on the amount and structure of F-actin filaments present in MNCs. In their study, the pretreatment of acutely isolated MNCs with cytochalasin-D, a compound that promotes actin depolymerization, reduced the mechanosensitivity index, and the opposite was observed with jasplakinolide, a cell-permeable actin polymerization agent. The authors concluded that osmosensory transduction relies on changes in the cell volume and also speculated about possible physical interaction of actin filament with stretch-inactivated cation channels (Zhang et al., 2007). In another respect, a study performed in the same year by Tobin and Ludwig (2007), revealed that induction of actin polymerization by jasplakinolide did not alter peptide release in the neuronal lobe during high-K^+^-stimulation, demonstrating that the axonal peptide release is not dependent on actin filaments remodeling. The gating model proposed assumes that during hypertonicity, the cell shrinkage would lead to a reorganization of the microtubules in a direct way, causing a compression force sufficient to change the channel conformation and induce its activation (Prager-Khoutorsky et al., 2014). This phenomenon was further linked to the modulation of TRPV channels in the MNCs (Ciura, 2006; Ciura et al., 2011; Moriya et al., 2015; Zaelzer et al., 2015). Therefore, those findings reinforce the idea that the ultrastructure of cytoskeleton elements has important functional roles in associating volume changes with plasma membrane events in the MNCs. Nevertheless, implicit in the mechanosensitive argument is the assumption that MNCs respond to steady changes in osmolality with a steady change in cell volume, i.e., these cells should present neither RVD nor regulatory volume increase (RVI). Therefore, it seems that the response of MNCs to osmotic challenges is more complex than those predicted by a simple osmometer.

Based on the assumption that water fluxes across the plasma membrane lead to changes in cell volume, elegant studies were performed combining voltage-clamp recording and soma volume measurements during variations imposed on the extracellular fluid osmolality (Oliet and Bourque, 1993; Zhang et al., 2007). Changes in osmotic pressure, induced by either hypertonic stimulus or hydrostatic pressure (applied through a patch pipette), resulted in decreased cell volume and activation of an inward current with a linear *IxV* relationship and reversal potential around −10 mV. These data suggest the involvement of voltage-independent and non-selective cation channels in the osmotransduction mechanism (Bourque, 1989; Oliet and Bourque, 1993, 1994). These responses occurred in the absence of changes in the extracellular Na^+^ concentration and were due to the cell volume-dependent regulation (Oliet and Bourque, 1993). In both cases, the conductance activated by both osmotic stress and hydrostatic pressure was entirely blocked by Gd^3+^ (Oliet and Bourque, 1996; Zhang et al., 2007), a potent but not exclusive blocker of stretch-inactivated channels (Elinder and Arhem, 1994; Hongo et al., 1997). In neurons of the cortex and hippocampus (Oliet and Bourque, 1993), the hypertonicity/hypotonicity and the negative/positive pressure also provoke volume changes, but these are not accompanied by variations in membrane conductance (Oliet and Bourque, 1993). These results support the hypothesis that intrinsic osmosensory transduction is a property of the MNCs themselves and that the mechanism involved seems to be basically a mechanical process.

Mechanical transduction by ion channels may occur in any cell, including red blood cells (for a review, see Sachs, 2015). Both stretch-activated channels (SACs) and stretch-inactivated channels (SICs) (Sachs, 2010) have been described in MNCs. Single-channel analysis revealed that hypertonicity-induced cell shrinkage increases the probability of SIC opening, consistent with the resting potential depolarization observed in whole-cell recordings (Bourque and and Oliet, 1997; Bourque, 1998; Zhang et al., 2007). At first glance, this evidence implies that RVI may be absent in these cells and that they lack a volume regulatory mechanism. Although the short-term (a few minutes) response of MNCs to hypertonicity is a decrease in cell volume, other processes seem to occur in the long run (tens of minutes to hours), including somatic hypertrophy of MNCs after hyperosmotic challenge (Hatton and Walters, 1973; Shah et al., 2014). Shah et al. (2014) reported a significant increase in the cross-sectional area of the cell after a considerable delay upon exposure of isolated MNCs to a *in vivo* hyperosmotic condition. This phenomenon is dependent on the exocytotic fusion of secretory vesicles and therefore is dependent on calcium entry in the cell and activation of phospholipase C. In line with the above results, Sato-Numata et al. (Sato-Numata et al., 2021) reported that AVP MNCs responded to a hyperosmotic challenge with both a membrane shrinkage and a massive release of the hormone, leading to a decrease in secretory volume. This latter phenomenon was impaired by tetanus toxin and blockers of T-type calcium channels, suggesting that it originates from vesicle fusion to the plasma membrane. They also found that the addition of flufenamic acid, which blocks voltage-gated Ca^2+^ channels (T and N types), prevents both the firing rate increase and the associated neuropeptide secretion. Under this experimental condition, the RVI mechanism became evident. According to the authors, RVI is hidden behind a substantial exocytosis, which occurs in a hyperosmotic stimulation and is dependent on the functioning of the Na^+^/H^+^ exchanger and of the Cl^−^/HCO_3_^−^ anion exchanger. As pointed out by Zhang and Bourque (2003), MNCs possess a significant membrane reserve, which allows them to blunt the membrane tension induced by osmotic perturbation. Would this be sufficient to ensure the maintenance of the stimulus-secretion coupling when the MNCs are exposed to a long period (hours to days) of hyperosmotic challenge, such as during prolonged water deprivation or salt-loading? As we discuss in the coming sections, under chronic dehydration, the MNCs experience dramatic transcriptomic remodeling, which includes changes in the expression of several genes encoding plasma membrane channels and transporters (Hindmarch et al., 2006; Greenwood M. P. et al., 2015; Johnson et al., 2015; Pauža et al., 2021). Another important adaptation is the increased complexity of the cytoskeleton array observed during sustained hyperosmolality (Barad et al., 2020; Hicks et al., 2020), whose crucial role for osmosensitivity was previously demonstrated by Prager-Khoutorsky et al. (2014), also discussed later in this review. Thus, the new molecular phenotype of the MNCs under chronic osmotic challenge might also help understanding how they are able to ensure the continuous AVP and OXT secretion, essential for survival during dehydration, despite the incessant plasma membrane insertion due to the vesicle fusion. Therefore, the fusion of membrane secretory vesicles added to the bilayer blunts the development of tension and masks the volume regulatory mechanism. These results may explain, at least in part, the absence of membrane tension for gating mechanosensitive channels and suggest another gating mechanism involving the cytoskeleton components, besides the classical bilayer-tension phenomenon. In this context, it is known that MNCs readily express a plethora of ion channels and transporters that may contribute to the complexity of the osmosensing mechanism.

### Genes, Channels, Transporters and Osmoregulation in the SON

Pauža and collaborators recently classified and catalogued all membrane transporters coded by mRNAs expressed in the SON of control rats. About 400 mRNAs coding membrane transporters and 220 mRNAs coding channels (or channel subunits) have been identified in the SON under isotonic conditions (see Figure 3 and Supplementary Figures 6 and 8 in Pauža et al., 2021). Therefore, in this section we focus on the discussion of key mRNAs that code channels and transporters (classified according to the International Union of Basic and Clinical Pharmacology, IUPHAR) in the SON, the functional relevance as well as the plastic response after dehydration.

As a brain nucleus with high metabolic demand and implicated in the synthesis and release of large amounts of peptides in the periphery and the CNS (Roy et al., 2021), it is not surprising that the genes mostly expressed in the SON encode proteins directly related to mitochondrial ATP synthesis and use by active transport processes, such as Na^+^/K^+^ ATPase and H^+^ ATPase. The most expressed mRNA of a cotransporter was *Slc22a17*, coding the protein BOCT1. This transporter has been described as highly expressed in the brain, mainly in neurons (Bennett et al., 2011). It was recently demonstrated that BOCT1 works as a membrane receptor for circulating lipocalin 2, a biomarker of acute and chronic renal injury (Jaberi et al., 2021). Hyperosmolality upregulates BOCT1 expression in collecting duct cell lines via CREB signaling, suggesting that this protein contributes to osmotolerance at the renal level (Jaberi et al., 2021). It was previously demonstrated that peripheral lipocalin 2 can cross the blood-brain barrier and act on the PVN neurons to modulate food intake (Mosialou et al., 2017). Thus, we can speculate that this transporter may also mediate some of the lipocalin 2 effects on the MNCs and/or participate in their osmosensory process. The second most expressed mRNA was *Slc1a3*, also known as GLAST1 (sodium-dependent glutamate/aspartate transporter), present in glial cells and shown to increase in the SON in conditions of heart failure (Potapenko et al., 2012) and salt loading (Choe et al., 2016). It also plays an important role in modulating the extracellular levels of glutamate in the SON to control the MNCs activity (Souza et al., 2020). In the control state SON, *Aqp4* is the highest expressed among mRNAs coding channels. As previously mentioned, astrocytes are rich in AQP4. Recently, it was demonstrated that AQP4 is required for the osmotically induced increase in neuronal activity of AVP-producing MNCs by mediating the retraction of astrocyte GFAP filaments (Wang et al., 2021). Next comes *Gja1*, which encodes connexin 43, important for intercellular communication among glial cells, mainly astrocytes (Duan et al., 2004). Furthermore, a heterotypic connexin 43/32 gap junction has been described between astrocytes and neurons in the SON. When this heterotypic gap junction was blocked by carbenoxolone, the FOS activation in the MNCs in response to salt overload was significantly reduced, indicating a key role played by connexin 43 in the astrocyte-MNC communication during hypertonicity-induced AVP secretion (Duan et al., 2004). The ion channel-related mRNA *Gabra1* coding for the gamma-aminobutyric acid type A receptor subunit alpha-1 is also highly expressed in the control SON (Pauža et al., 2021). MNCs isolated from adult rat brains respond to GABA, functionally demonstrating that those neurons express GABA receptors (Oliet and Bourque, 1992). It was shown that the gamma-aminobutyric acid type A receptor subunit alpha-1 and -2 are co-expressed with the gamma 2 subunit in MNCs, suggesting they may express GABAA receptors with benzodiazepine type 1 and 2 pharmacological properties (Fenelon and Herbison, 1995). Despite having inhibitory effects in most neurons in the adult brain, there is strong evidence indicating that GABA acting via GABAA receptors is excitatory in the adult AVP-producing MNCs under basal condition (Haam et al., 2012). In sequence are mRNAs coding the glutamate-activated NMDA and AMPA channel subunits, *Grin1* and *Gria2* respectively, highly expressed in the SON (Pauža et al., 2021). The stimulatory effect of l-glutamate on MNCs’ electrical activity and hormonal secretion was demonstrated both *in vitro* (Richard and Bourque, 1995; Sladek et al., 1995) and *in vivo* (Xu and Herbert, 1998; Onaka and Yagi, 2001). It was further shown that the activity of AVP- and OXT-producing neurons is differentially modulated by NMDA and AMPA l-glutamate receptors. These results suggest that the AVP neurons are influenced by both NMDA and AMPA receptors, while the OXT neurons seem to be mainly regulated by AMPA receptors (Nissen et al., 1995; Richardson and Wakerley, 1997). By using intra-SON microinjection, Busnardo et al. (2012) demonstrated that AVP and OXT secretion is mediated by AMPA receptors. In this context, it has recently been shown in *in vivo* experiments that hyperosmolality and hemorrhage affect AVP secretion through activation of NMDA and AMPA receptors (Vilhena-Franco et al., 2018; Busnardo et al., 2021; Dos-Santos et al., 2022). Those findings indicate that the MNCs are subject to the influence of both l-glutamate and GABA activated channels and that their close interaction with astrocytes is essential to integrate the osmoreception process in the SON.

**FIGURE 1:**
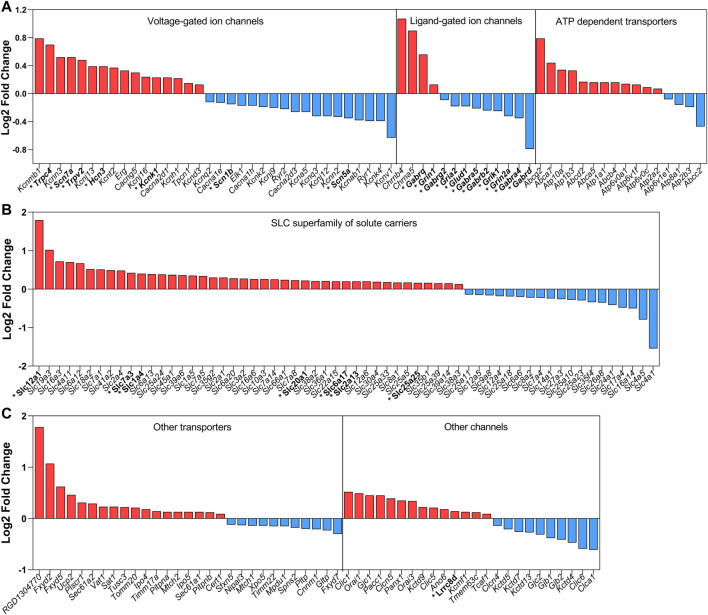
Summary of the mRNA coding channels and transporters found to be changed by water deprivation for 3 days. Data obtained with the RNAseq approach by Pauža et al. (2021). In red are the upregulated mRNAs and in blue are the downregulated mRNAs. In this global analysis, we found coding mRNAs significantly and differentially regulated for 34 voltage-gated channels (16 up and 18 down), 13 ligand-gated ion channels (4 up and 9 down), 24 other ion channels (14 up and 10 down), 61 SLC superfamily of solute carriers (42 up and 19 down) and 29 of other transporters (18 up and 11 down). The mRNAs coding channels and transporters that we discuss in the present review are highlighted with an asterisk and in bold.

Additionally, Pauža et al. (2021) have also demonstrated that the mRNA expression of each channel and transporter was changed in the SON of rats exposed to 72 h of water deprivation. Since it is practically impossible to discuss every gene upregulated/downregulated in the SON after osmotic stress, a global view can be seen in Figure 1. This figure summarizes the data regarding all mRNA coding channels and transporters found to be changed by 3 days of water deprivation, using the RNAseq approach (Pauža et al., 2021). The great majority of those channels and transporters have not yet been characterized with respect to their protein expression and/or function in the MNCs and/or their associated glial cells in the SON.

**FIGURE 2:**
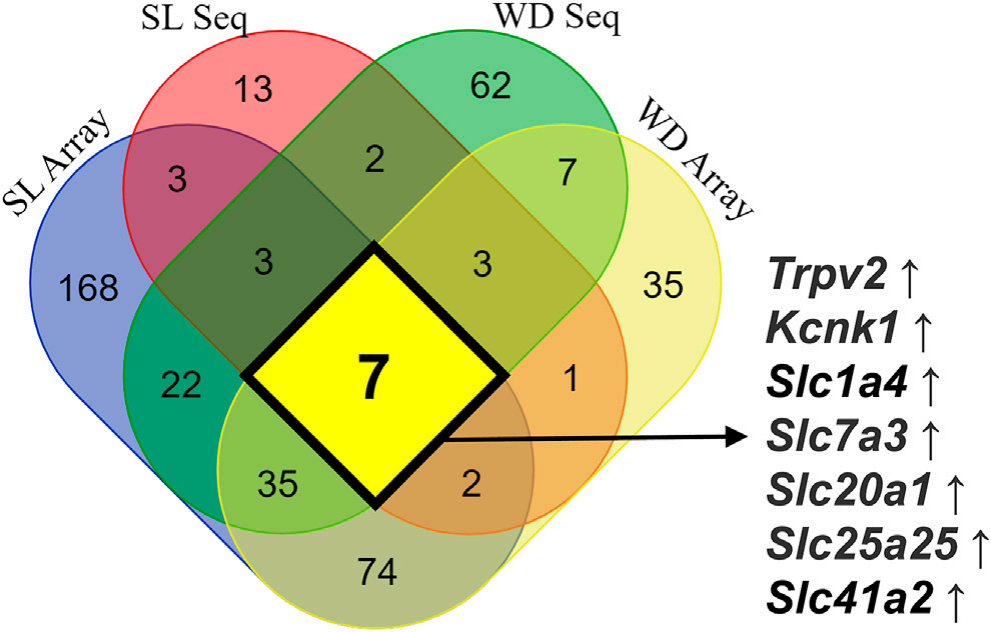
Integrated data on the expression of mRNA coding ion channels and membrane transporters in the SON in response to long-term exposure to hyperosmolality in rats. Microarray data obtained from water-deprived (WD) rats are from Hindmarch et al. (2006), microarray information from WD and salt loading (SL) rats is from Greenwood M. P. et al. (2015), RNAseq data obtained from SL rats are from Johnson et al. (2015), and finally data on RNAseq obtained from WD rats are from Pauža et al. (2021). This integrated analysis revealed seven core genes coding channels and transporters consistently upregulated by sustained hyperosmotic stimulation: *Trpv2, Kcnk1, Slc1a4, Slc7a3, Slc20a1, Slc25a25,* and *Slc41a2*.

When animals are subjected to a prolonged dehydration time, the whole transcriptome of the SON is remodeled to guarantee adequate response of the MNCs to the imposed condition. Murphy’s group has used the transcriptome approach in the recent years to describe the remodeling of the SON global gene expression in dehydrated animals and to discover new players regulating the MNCs activity-synthesis-secretion response, such as CREB3L1 (Greenwood M. et al., 2015), CAPRIN2, (Konopacka et al., 2015a), RASD1 (Greenwood et al., 2016) and NKCC2 (Konopacka et al., 2015b). However, except for *Slc12a1*, coding NKCC2, and a few other new genes discovered to be regulated by hyperosmolality in the SON, most of the membrane transporters and channels described have not yet been investigated regarding their role in osmosensitive processes. To summarize the data related to the expression of membrane transporters and channels in the SON in response to prolonged hyperosmolality in rats, we have integrated data from four publications revealing new possible players in the regulation of MNCs’ electrophysiological response. While Hindmarch et al. (2006) and Greenwood M. P. et al. (2015) used the microarray technology to analyze the SON transcriptomic response, Johnson et al. (2015) and Pauža et al. (2021) took advantage of the next generation RNAseq technique. Furthermore, Hindmarch et al. (2006) and Pauža et al. (2021) used 3 days of water deprivation to stimulate the SON, while Johnson et al. (2015) used 5 days of salt loading (hypertonic saline as the only fluid available), and Greenwood et al. (Greenwood M. P. et al., 2015) compared the SON transcriptomic response to 3 days of water deprivation or 7 days of salt loading. Water deprivation and salt loading can both increase *Avp* and *Oxt* mRNA expression and peptide secretion. However, while water deprivation is associated with a decrease in the extracellular volume and activation of the peripheral renin-angiotensin system, prolonged salt loading has no significant effects on extracellular volume and inhibits the activity of the peripheral renin-angiotensin system (Greenwood M. P. et al., 2015). Thus, despite the methodological caveats among these works and the important physiological differences between water deprivation and salt loading models, the prolonged hyperosmotic response of the MNCs is conserved. Those data show that the expression of seven core genes coding channels and transporters was increased: *Trpv2, Kcnk1, Slc1a4, Slc7a3, Slc20a1, Slc25a25,* and *Slc41a2* (Figure 2).

**FIGURE 3:**
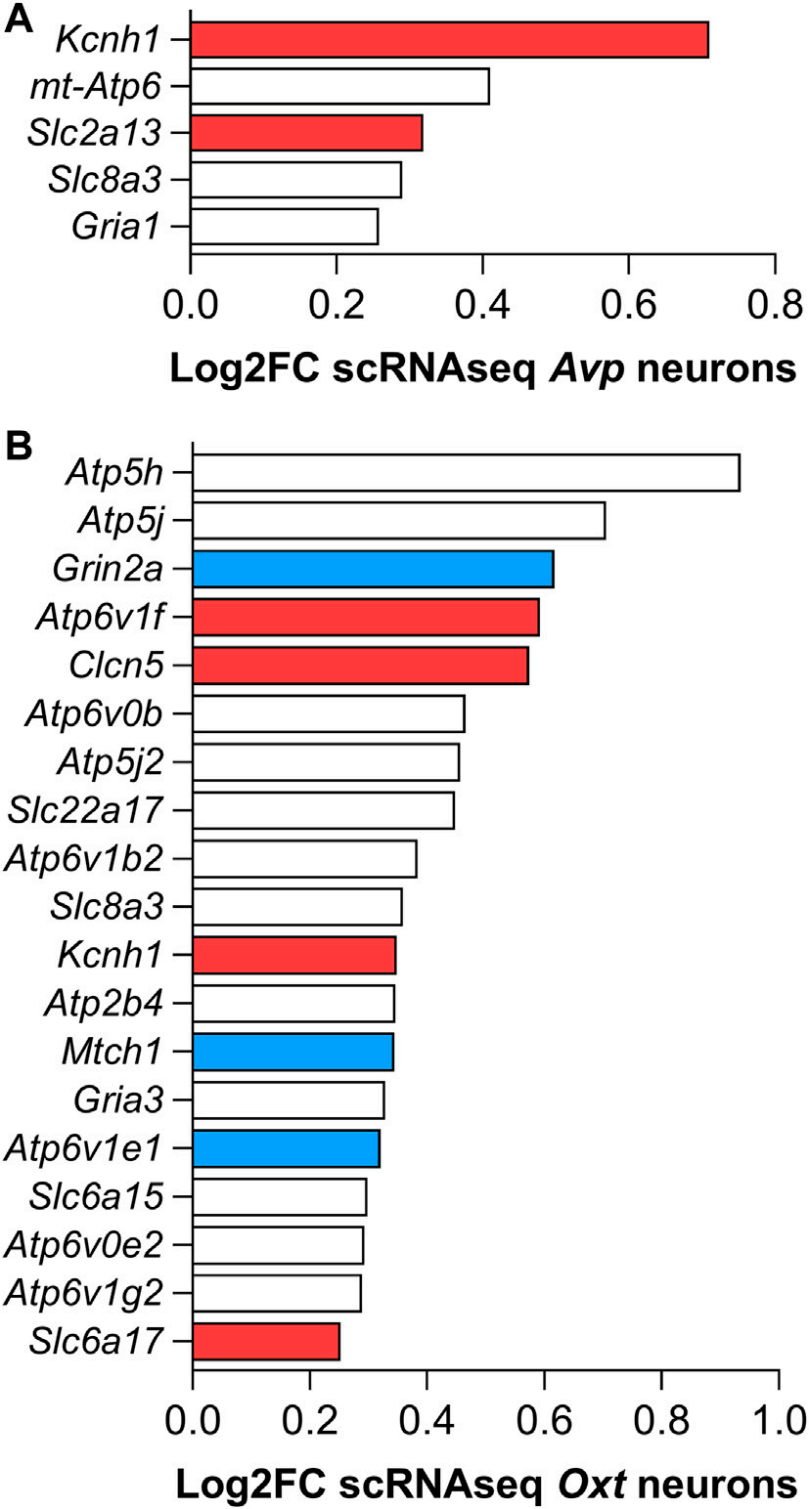
Set of mRNAs coding channels and membrane transporters positively enriched in **(A)** AVP- and **(B)** OXT-producing hypothalamic mouse neurons found with the single-cell RNAseq transcriptome analysis by Romanov et al. (2020). Red colored bars refer to the genes that, besides being positively enriched on AVP- and/or OXT-producing neurons in mice, are also up-regulated by 3 days water deprivation in the SON of rats. Blue colored bars refer to down-regulated genes (Pauža et al., 2021). White bars refer to genes also regulated in the SON but not discussed in this review.

Over the last few years, the development of the RNAseq technique has also allowed a deeper examination of specific genes enriched in specific cell populations. In this way, single-cell transcriptome data have revealed a set of genes positively enriched in AVP- and OXT-producing neurons along with hypothalamic development in mice (Romanov et al., 2020). The results regarding positively enriched genes that encode channels and transporters in both *Avp* and *Oxt* neurons are summarized in Figure 3.

Based on these results, in the following paragraphs we emphasize the current literature, indicating the possible roles played by some membrane ion channels and transporters on the osmosensitivity and electrophysiological activity of MNCs.

### TRPV Channels

In 1997, a gene encoding OSM-9, which is related to aversive behavior of *Caenorhabditis elegans* to strong hypertonic environments, was shown to be required for mechanosensation. This gene encodes a predicted protein with structural similarities to the mammalian transient receptor potential vanilloid (TRPV) (Caterina et al., 1997; Colbert et al., 1997; Lee et al., 2016)*.* Their molecular structure encompasses six transmembrane domains and a three to five multiple ankyrin-binding sequence repeats in the N-terminal and a large C-terminal intracellular domain (Liedtke and Kim, 2005). It has been demonstrated that TRPV1 participates in the mechanoelectrical transduction of MNCs in response to short-term hyperosmolality. In mice MNCs, the cationic conductance induced by hypertonicity is blocked by ruthenium red, a non-selective antagonist of TRPV channels (Sharif-Naeini et al., 2006). The same pattern of response was also observed in neurons from the OVLT (Ciura, 2006; Ciura et al., 2011). Intriguingly, HEK 293 cells heterologously expressing the classical TRPV1 channel did not respond to hypertonic stimulation (Liedtke et al., 2000). Although *Trpv1* was detected in MNCs by reverse transcription-polymerase chain reaction (RT-PCR), no signals for the N terminus of Trpv1 (exons 2–6) were detected in the SON (Sharif-Naeini et al., 2006). Immunohistochemistry experiments confirmed this result, showing immunoreactivity to TRPV1 when MNCs were probed with an antibody against the C-terminal but not against the N-terminal (Sharif-Naeini et al., 2006). The N-terminal region absent in MNCs represents a portion containing capsaicin-binding sites (Caterina et al., 1997), and explains why these cells’ activity did not change when exposed to capsaicin (Sharif-Naeini et al., 2006), a selective agonist of TRPV1. Additionally, neurons isolated from mice lacking *Trpv1* lost their ability to respond to increases in osmolality, indicating that *Trpv1* encodes a potential osmosensory channel in the SON (Sharif-Naeini et al., 2006). Taken together, these results lead to the conclusion that TRPV1 channels in MNCs have a particular molecular structure and that one or more *Trpv1* splice variants seem to be necessary to encode a capsaicin-insensitive channel (Sharif-Naeini et al., 2006; Moriya et al., 2015).

Alternative splicing is considered to be one of the major sources of the functional diversity of proteins (Johnson et al., 2003). To date, four TRPV1 splice variants containing modifications in the N-terminal intracellular domains have been described: 1) vanilloid receptor 5′ splice variant (VR.5′sv), which was the first variant reported and characterized regarding the lack of exons one to four and 7 (Schumacher et al., 2000); 2) TRPV1 beta (TRPV1b), which contains a modification in the N-terminal encoded by exon seven resulting in the loss of 30 nucleotides (Wang et al., 2004); 3) TRPV1var, which lacks exon 1, part of exon 16 and presents a failure in the splice out of intron 5 (Tian et al., 2006); 4) stretch-inhibitable nonselective cation channels (SICs) have identical trans-membrane-spanning domains as VR.5`sv. The C-terminal of SIC diverges from TRPV1 and VR.5’sv. It was demonstrated that the C terminal of SIC is derived from two related but independent genes (Schumacher et al., 2000; Xue et al., 2001; Tian et al., 2006). Xue et al. (2001) found that human and mouse TRPV genes share an extensive sequence homology, but only through exon 15. The remaining sequences encoding the proposed C-terminal domain of SIC matched the gene encoding vanilloid receptor-related–osmotically activated channel (VROAC, now called TRPV4).

Three out of four variants were identified in the SON by Moriya et al. (2015): full-length *Trpv1*, *Trpv1b* and SIC. Although MNCs express *Trpv1* mRNA, able to encode the full-length TRPV1, including the 36 amino acids essential to capsaicin action, the isolated MNCs did not respond to capsaicin (Sharif-Naeini et al., 2006; Moriya et al., 2015). Unlike Moriya and collaborators (2015), Zaelzer et al. (2015), using PCR cloning, isolated *Trpvdn*, a cDNA containing an open reading frame encoding all of 5–15 exons of *Trpv1*. Transfection of *Trpv1dn* into HEK293 cells induced the expression of a 58 Kd protein (truncated ΔN-TRPV1). This protein is significantly smaller than the full-length TRPV1, but sufficient to make the cell sensitive to hypertonicity. Most important, the expression of *Trpv1dn* in isolated MNCs from *Trpv1* knockout mice rescued the neurons’ ability to respond to osmotic stimuli.

An *in vitro* study demonstrated that the C-terminus of ΔN-TRPV1 physically interacts with microtubules (Goswami et al., 2004, 2007), suggesting that cytoskeleton elements may be part of the mechanism of channel activation. As described by Prager-Khoutorsky et al. (2014), microtubules have a unique array in the MNCs. Therefore, a proposed gating model assumes that during acute hypertonicity, cell shrinkage leads directly to reorganization of the microtubules, causing a compression force sufficient to change the channel conformation and induce its activation (Prager-Khoutorsky et al., 2014). At the molecular level, an elegant study conducted by Fisher´s group, showed that activation of ΔN-TRPV1 also involves phospholipase C (PLC) (Bansal and Fisher, 2017). Additionally, PLCδ1 knockout mice display an impaired electrophysiological response to increases in extracellular osmolality (Park et al., 2021). The idea is that PLCδ1 activated by Ca^2+^ influx during osmotically evoked MNCs action potential drives a positive feedback loop leading to a stronger activation of ΔN- TRPV1 (Park et al., 2021).

In spite of this compelling molecular and functional evidence about the role of ΔN-TRPV1 in MNCs’ osmosensitivity, we should note that *Trpv2* showed the highest level of expression among the mRNAs encoding TRPV channels in the SON of rats in the control condition (Pauža et al., 2021). Furthermore, *Trpv2* mRNA is consistently increased in response to chronic hyperosmolality in this nucleus (Figure 2). Besides this, TRPV2 protein is preferentially expressed in the MNCs of both the SON and PVN (Wainwright et al., 2004; Nedungadi et al., 2012b). The TRPV2 was also demonstrated to have its expression increased at both mRNA and protein levels in parallel with the increased levels of AVP heteronuclear RNA and protein levels in both SON and PVN of rats submitted to chronic bile duct ligation (Nedungadi et al., 2012a). This animal model is widely used to activate MNCs’ AVP hypersecretion, culminating in a secondary plasma hypoosmolality (Balapattabi et al., 2020). Studies of peripheral osmosensitive cells, such as vascular aortic smooth muscle cells, treated with *Trpv2* antisense oligonucleotides demonstrated reduced TRPV2 expression, suppression of the nonselective cationic current and elevation of the intracellular Ca^2+^ concentration in response to hypotonic stimulation (Muraki et al., 2003). Later, it was also demonstrated that TRPV2 is critical for the osmotic response in skeletal muscle fibers. The TRPV2 dominant-negative showed lower membrane depolarization, a reduced intracellular Ca^2+^ concentration and RVI response. The authors further demonstrated that the response of TRPV2 depends on the sequential activation of STE20/SPS1-related proline/alanine-rich kinase (SPAK) and NKCC1 cotransporter (Zanou et al., 2015).

Similarly to TRPV1 (Prager-Khoutorsky et al., 2014), TRPV2 also interacts with cytoskeleton structures, mainly with actin filaments, and its activation depends on rearrangements of the actin filaments in response to mechanical stimulation (Sugio et al., 2017; Yadav and Goswami, 2020). The cortical actin filaments of the MNCs are uniquely organized, and their increased array complexity after salt-loading treatment seems to be involved in the elevated osmo-responsiveness following sustained intracellular dehydration (Barad et al., 2020). Hence, TRPV2 might have an actin filament interaction-dependent role in the MNCs’ volume-electrical activity coupling efficiency, maintenance and/or potentiation during prolonged dehydrated state.

Due to the lack of functional evidence about the role of TRPV2 in the MNCs’ osmosensitivity and the fact that smooth and skeletal muscle cells are not osmosensitive in the same way as MNCs, we can only speculate about a possible role of this channel in the control of the intrinsic osmosensitivity and electrophysiological properties. Future studies need to be carried out to clarify if TRPV2 participates in the osmosensitivity process of MNCs, especially under chronic hyperosmotic stimulation. A point that still needs to be elucidated for both TRPV1 and TRPV2, or even other mechano-gated channels, is whether the traction force induced by cytoskeleton elements occurs only in the C-terminal end, or it is somehow transferred to the lipid bilayer, resulting in a membrane-tension-complex for activation.

Other potential osmoreceptors are the canonical transient receptor potential (TRPC) 5 and 6. Although not investigated in MNCs, TRPC5 (Jemal et al., 2014) and TRPC6 (Wilson and Dryer, 2014) channels were also shown to be sensitive to mechanical and osmotic stress in heterologous expression systems, but different from TRPVs expressed in the MNCs, TRPC5 and 6 are sensitive to hypotonic stress. These canonical members, especially TRPC4, were recently linked to neuronal exocytosis by mediating additional Ca^2+^ influx followed by mGluRs activation (Jeon et al., 2021). In MNCs, Nedungadi and Cunningham (2014) and Pauža et al. (2021) identified the presence and increased gene expression of *Trpc4* after 48 h of water deprivation, suggesting the participation of this channel in osmoregulation.

In summary, although the results described above convincingly point to ΔN-TRPV1 as the main molecular mechanism coupling hyperosmolality to electrical responses in the MNCs, a significant number of studies have shown that modulation of MNCs’ activity during osmotic challenges relies on several additional factors, including a variety of other ion channels and transporters (Stern and Ludwig, 2001; Pires da Silva et al., 2016; Bansal and Fisher, 2017; Ferreira-Neto et al., 2021). The list of genes up and downregulated in the SON in response to water deprivation reinforces this argument (Figure 1). Therefore, depending on the time window of the osmotic challenge, MNCs might recruit distinct channels and transporters to collectively coordinate their electrophysiological response to extracellular osmolality.

### Potassium Channels

Among the mRNAs coding for ion channels is *Kcnk1, which* is consistently upregulated by hyperosmolality. This gene is responsible for coding the potassium two pore domain channel subfamily K member 1, also known as TWIK1. Each subunit of the genes coding the channels of this family is responsible for the expression of the two-pore loop domains that form one ion pathway selective to K^+^ (Kwon et al., 2021). It has been demonstrated that these channels can also be modulated by temperature and osmolality (Talley et al., 2003). The TWIK1 is expressed at the brain level in both neurons and astrocytes (Kwon et al., 2021). Single-channel recordings from isolated MNCs have also identified some background (leak) K^+^ channels (TREK-1, TREK-2, and a novel TREK), which are sensitive to membrane stretching (Han et al., 2003). Additionally, voltage-gated K^+^ channels also seem to play a role in the osmotically evoked MNCs’ activity by shaping their firing pattern (Zhang et al., 2009). Electrophysiological recording in acutely isolated MNCs showed that hypertonicity (325 mOsm/KgH_2_O) induces an outward current whose equilibrium potential is near to that expected for K^+^ (Zhang et al., 2009). The authors hypothesized that it is mediated by KV7/M-type channels, since the outward hypertonic sensitive-current was blocked by XE991 and enhanced by retigabin, a selective inhibitor and activator of M-type K^+^ currents, respectively. The contribution of this current to osmosensitivity of MNCs is unusual, but could contribute to the transition from continuous to burst activity (Zhang et al., 2009).

Single-cell RNAseq revealed that mRNA coding *Kcnh1* is positively enriched in both *Avp*- and *Oxt*-producing MNCs (Figure 3) (Romanov et al., 2020). Furthermore, our SON RNAseq data also demonstrate increased expression of this gene after 3 days of water deprivation (Pauža et al., 2021). Kcnh1 codes the K^+^ channel KCNH1, also known as Ether-à-go-go1 (Eag1), a voltage-gated K^+^ channel family member, with four alternative transcripts identified in the human brain (Ramos Gomes et al., 2015). Recently, this channel was identified in the presynaptic terminals of parallel fiber–Purkinje cells, acting as a modulator of presynaptic action potentials and regulating the Ca^2+^ influx and neurotransmitter release during high-frequency burst firing. Furthermore, the authors found no changes in the excitability or action potential shape at the cell somata (Mortensen et al., 2015). Thus, despite no characterization of the KCNH1 functional protein expression in the MNCs or at the neurohypophysial terminal so far, this channel is another candidate for participation in the control of MNCs’ electric activity and/or in the neuropeptide secretion as a response to hyperosmotic stimulus. However, future functional studies need to be carried out to clarify its possible role on MNCs’ physiological responses.

### Sodium Channels

Even in the absence of osmotic disturbances, changes in Na^+^ concentration also activate inward currents in MNCs, which are a consequence of changes in the Na^+^ driving force and the relative membrane permeability to this ion (Bourque, 1989). Physiologically, this phenomenon becomes significant when considering that *in vivo*, systemic osmotic pressure disturbances are accompanied by parallel changes in the Na^+^ concentration in the extracellular fluid. Therefore, it is reasonable to assume that non-voltage and/or voltage-dependent Na^+^ channels may act as sensors to detect increments in Na^+^ concentration in the SON, contributing to the modulation of MNCs' electrical properties during osmotic stress.

Non-voltage dependent channels, particularly the epithelial Na^+^ channel (ENaC), were identified in the SON by immunocytochemistry (Amin et al., 2005) and their functionality was ascertained by Teruyama et al. (2012), by associating pharmacology and electrophysiological recordings in brain slice preparations. They demonstrated that ENaC has a significant influence on the resting membrane potential of MNCs, since its blockage, by amiloride or benzamil, resulted in hyperpolarization of the membrane potential and cessation of action potential firing (Teruyama et al., 2012). Additionally, the expression and activity of ENaC are influenced by a high sodium diet (Garty and Palmer, 1997). A diet based on high NaCl intake for 7 days increased the expression of the channel (Sharma et al., 2017) and caused a depolarization of the membrane potential of MNCs, which was restored to basal levels by benzamil (Teruyama et al., 2012). Therefore, the steady-state Na^+^ leak current mediated by ENaC also seems crucial for controlling MNCs’ excitability and firing pattern, which ultimately controls hormone secretion during osmotic imbalance induced by changes in extracellular Na^+^ concentration.

Besides this, several voltage-dependent sodium channels also seem to control MNCs’ activity during osmotic challenge. Transcriptome analysis of the SON of water-deprived rats demonstrated an increase in the expression of *Scn7a* and a decrease in *Scn1b* and *Scn5a* mRNAs (Figure 1) (Pauža et al., 2021). Additionally, after 10 days of salt-loading, two Na^+^ channel α subunits and the corresponding β1 and β2 subunits were shown to be upregulated, both at the mRNAs and protein levels, suggesting a physiological role of these channels (Black et al., 2013, 2014). These molecular findings point to the distinct regulatory effects of water deprivation and salt-loading on sodium voltage-gated ion channels.

The presence and functionality of these sodium channels in the membrane, whose genes were upregulated, were confirmed by electrophysiological detection of a transient Na^+^ current (Tanaka et al., 1999). A significant difference was found for isolated cells from salt-loaded compared with those from euhydrated animals. A salt-loading diet induced a large transient Na^+^ current, and this response became more evident by applying slow-ramp depolarization voltage protocols. It was possible to observe an even more significant increase in amplitude and density of the persistent Na^+^ current evoked at subthreshold potentials (Tanaka et al., 1999). As far as we know, the functional impact of sodium voltage-gated ion channels’ plasticity in MNCs following water deprivation is still unknown.

Assuming that non-voltage and voltage-dependent sodium channels are also part of the mechanisms controlling the excitability of MNCs when the extracellular fluid homeostasis is disturbed by increases in extracellular Na^+^, an intriguing question arises: Are MNCs subject to a dual control system, i.e., how are sodium mechanisms integrated with those derived from changes in osmotic pressure only?

It was previously demonstrated *in vivo* that changes in plasma osmolality and extracellular sodium concentration could individually modulate the release of AVP in cells of goats, sheep and dogs (Olsson and Kolmodin, 1974; McKinley et al., 1978; Thrasher et al., 1980). Assuming that the release of neuropeptides is directly correlated with the firing rate of MNCs (Poulain and Wakerley, 1982), these cells are expected to present different electrical behavior for each kind of stimulus. In fact, in isolated MNCs obtained from coronal hypothalamic slices of rats, Voisin et al. (1999) observed that hypertonic stimulation with excess NaCl is significantly more potent to depolarize the membrane potential than an equivalent osmotic stimulus induced by mannitol. Additionally, in the absence of osmotic perturbation, MNCs are still efficient in changing their intrinsic activity during manipulation of extracellular sodium concentration. The inward current induced by isoosmotic increases in the extracellular sodium concentration (osmolality constant at 290 mOsm/kgH_2_O) or applications of hypertonic mannitol (+30 mOsm/kgH_2_O, with extracellular sodium constant at 125 mM) were present in both situations. However, only osmotically evoked currents induced by mannitol were able to change the membrane conductance of MNCs. Thus, associated with the fact that sodium currents were successfully inhibited by Gd^3+^, a blocker of SIC/ΔN-TRPV1 channels, the authors suggested that the Na^+^ sensitive current in MNCs flows through mechanosensitive cation channels in a coincident manner.

Despite all these facts, it was demonstrated that TRPV1 knockout rats show normal AVP secretion and thirst behavior in response to hypernatremia (Tucker and Stocker, 2016), suggesting that other channels are involved in the osmosensation process. Several sodium channel genes are expressed in the SON, some of which are significantly regulated during osmotic challenge (Hindmarch et al., 2006; Konopacka et al., 2015a; Johnson et al., 2015; Pauža et al., 2021). Therefore, since the electrical activity itself may modulate the expression of sodium channels, as previously suggested (Offord and Catterall, 1989), the remodeling of the electrogenic machinery may also be a crucial phenomenon for MNCs to maintain efficient neuropeptide secretion during a chronic osmotic disturbance.

Glial cells also substantially contribute to the control of MNCs’ activity during osmotic challenge. It has been shown that taurine is an essential gliotransmitter released to inhibit the firing rate of MNCs during hypotonic disturbances (Decavel and Hatton, 1995; Choe et al., 2012). Additionally, there is experimental evidence showing that the expression of the protein c-FOS (a marker of cell activity) in SON astrocytes, induced by a hyperosmotic stimulation, precedes the increased expression of the same protein in MNCs (Ludwig et al., 1996; Yuan et al., 2009). Interestingly, c-FOS protein in MNCs was inhibited by fluorocitrate, a glial metabolic inhibitor (Yuan et al., 2009).

Mice astrocytes express the sodium channel sensor (Na_x_) and are the primary site of sodium-level sensing in SFO (Iadecola, 2007; Hiyama and Noda, 2016), suggesting a homeostatic control mechanism of the SON by these cells (Watanabe et al., 2006). However, an important point to be considered is that mice and rats show differences in the Na_x_ expression, implying that the mechanism involved in Na^+^ detection in this nucleus may be specific to each rodent species. According to Nehmé et al. (2012), Na_x_ is more strongly expressed in rats than in mice. This evidence may explain, at least in part, why Na_x_ deficient mice present normal AVP secretion after dehydration (Nagakura et al., 2010). Therefore, neighboring cells, like astrocytes, may constitute active elements involved in response to hypertonic stimulation. However, little is known about how these cells drive these responses and whether they are necessary to trigger osmoregulation-related cell behavior.

### HCN Channels and Its Interaction With Nitric Oxide

An important family of channels related to the electrophysiological properties of MNCs is the hyperpolarization-activated cyclic nucleotide-gated potassium channel (HCN) (Ghamari-Langroudi and Bourque, 2000; Pires da Silva et al., 2016). Four subtypes of HCN channels (HCN1-HCN4) have been identified, and all of them are expressed in MNCs, with HCN-3 and HCN-4 mRNAs having the highest expression levels (Monteggia et al., 2000; Notomi and Shigemoto, 2004; Pires da Silva et al., 2016). These channels are involved in basic neuronal properties in the SON, including regulation of the resting membrane potential and spontaneous firing rate (Pires da Silva et al., 2016), besides promoting excitatory drive, contributing to the phasic and tonic firing in these cells (Ghamari-Langroudi and Bourque, 2000). HCN channels are endogenously inhibited by NO, since the blockage of nNOS by l-NAME results in a significant increase in the macroscopic current (I_h_) carried by these ion channels (Pires da Silva et al., 2016). In the same study, voltage-clamp experiments, performed in brain slices and *in situ* preparations, revealed that hypertonicity significantly increases the I_h_ current, which is directly correlated with increased release of neuropeptides. The effect of hypertonicity on HCN channels is also enhanced by l-NAME, indicating the existence of nitrergic inhibition even during hyperosmotic stress. Besides this, hypotonicity changed neither the instantaneous nor the steady-state I_h_ current, ruling out this pathway during this type of stimulation (Pires da Silva et al., 2016). Despite being upregulated at the mRNA level, indicating a possible increase in protein expression, in hypoglossal motoneurons, for example, the HCN channels are also functionally highly regulated by post-translational modification, such as S-nitrosylation (Wenker et al., 2012). The nitric oxide produced by the action of the enzyme nitric oxide synthase (NOS) was reported to be the main factor for S-nitrosylation in proteins (Jaffrey et al., 2001).

Interestingly, the transcriptome data show that the mRNA for the neuronal isoform of NOS (nNOS, coded by the gene *Nos1*) is the most upregulated enzyme in the SON after water deprivation (Pauža et al., 2021). This neuronal isoform is the most expressed type in the SON (Bhat et al., 1996), and it has been directly correlated with changes in plasma osmolality (Vincent and Kimura, 1992; Kadowaki et al., 1994; Ueta et al., 1995; Srisawat et al., 2004; Yamova et al., 2007). Following nNOS activation, l-arginine is oxidized, producing equimolar concentrations of NO and l-citrulline (Bush et al., 1992). This reaction occurs in the MNCs themselves (da Silva et al., 2013), and NO seems to act through autocrine, paracrine, and/or endocrine pathways (Calabrese et al., 2007; Bahadoran et al., 2020). Accumulated data show that the endogenously produced NO has an inhibitory role on MNCs (Liu et al., 1997; Ozaki et al., 2001; Stern and Ludwig, 2001; Ventura et al., 2008; da Silva et al., 2013; Reis et al., 2016). This inhibition can happen by two distinct routes: 1) by influencing synaptic plasticity, and 2) by directly modifying the function of a wide range of proteins, including those operating via ion channels.

Regarding synaptic plasticity, it was demonstrated that sodium nitroprusside (SNP, a donor of NO) increased the frequency of spontaneous inhibitory postsynaptic currents but did not affect the excitatory ones mediated by non-NMDA glutamate receptors (Ozaki et al., 2001). The same pattern of response was also demonstrated in measurements of GABA_A_ miniature inhibitory postsynaptic currents, which reflect the activity of a single synaptic site (Stern and Ludwig, 2001). Therefore, according to the authors, NO has an indirect paracrine effect on the activity of MNCs by potentiating GABAergic inputs. Although there is no doubt that NO potentiates GABAergic synaptic input, there are also findings of direct action of NO on the MNCs, independent of synaptic inputs. In this case, the HCNs, working as intrinsic peacemakers in various cell types, seem to be the NO target.

NO has considerable ability to interact with cysteine residues, forming nitrosothiol adducts. These interactions influence several cellular processes by altering the function of proteins via reversibly binding to thiol groups of cysteine residues (Hess et al., 2005). This association results in a more stable complex, S-nitrosothiol, which prolongs the biological activity of NO (Ahern, 2002). Pretreatment of brain slices of the SON with N-ethylmaleimide, a cysteine oxidant, prevents l-arginine’s nitrergic inhibition of I_h_ currents. Additionally, denitrosylation induced by ascorbate resulted in a significant increase in I_h_ currents, revealing an endogenous nitrosylation of HCN channels and a cGMP independent signaling pathway of NO in MNCs in basal condition and during a hypertonic challenge (Pires da Silva et al., 2016). Thus, the inhibitory effects of NO on GABAergic inputs and HCN channels occur synergistically to control the MNCs’ excitability, and consequently the release of neuropeptides. During short-term osmotic pressure perturbation, nitrergic inhibition is still present, indicating that NO works by self-regulating the neuroendocrine system to avoid neuronal hyperexcitability and neuronal cell death.

Based on the results described above, we can say that because nNOS activation depends on intracellular calcium, ΔN-TRPV1 (and potentially other channels of the TRPV family, such as TRPV2) would represent an important pathway for Ca^2+^ entry and NO synthesis during hyperosmotic perturbation. Additionally, since the activation of classical TRPV1 by NO-cysteine S-nitrosylation was also confirmed to occur in cysteine 553 and 558 (Yoshida et al., 2006), and ΔN-TRPV1 conserved these residues (Zaelzer et al., 2015), it is reasonable to assume that NO can also promote S-nitrosylation of ΔN-TRPV1 and other membrane channels and transporters controlling MNCs’ excitability and hormone secretion. However, whether it acts only through HCN channels or involves other ion channels or intracellular signaling in MNCs is still unknown. For other aspects related to NO’s effect on MNC activity, please see Ruginsk et al. (2015).

### Transporters

Evidence about the participation of transporters in osmoregulation has also emerged from four publications (Hindmarch et al., 2006; Greenwood M. P. et al., 2015; Johnson et al., 2015; Pauža et al., 2021). After prolonged hypertonicity, five of the genes were identified that consistently increased mRNA for membrane transporters in the SON. 1) *Slc1a4*, coding the alanine/serine/cysteine/threonine transporter 1 (ASCT1); 2) *Slc7a3*, coding the cationic amino acid transporter 3 (CAT3), involved in the uptake of the amino acids ornithine, lysine and arginine in a sodium-independent manner; 3) *Slc20a1*, coding a sodium-dependent phosphate transporter 1 (PiT1); 4) *Slc25a25*, coding the mitochondrial phosphate carrier 3 (APC3); and 5) Slc41a2, coding a plasma membrane magnesium transporter (Figure 2). CAT3 is specifically present in neuronal cells (Hosokawa et al., 1999), while PiT1 is widely expressed in neurons, astrocytes and vascular endothelial cells (Inden et al., 2016). Finally, the mitochondrial phosphate carrier 3 is expressed in the inner mitochondrial membrane and works as a Ca^2+^-regulated shuttle for Pi and ATP-Mg^2+^, helping to control the mitochondrial metabolism (Anunciado-Koza et al., 2011).

The NKCC2, long known to be expressed in the apical membrane of the thick ascending limb of Henle’s loop, was found to be the most intensively upregulated gene coding for transporters of the SLC superfamily after water deprivation in the SON (Pauža et al., 2021). This transporter belongs to the subfamily of cation-chloride electroneutral cotransporters that couple the movement of sodium, potassium and chloride across the plasma membrane. Under physiological conditions, they have small or no impact. However, when cells are subjected to hyperosmotic shrinkage, their participation seems fundamental to restoring homeostasis. In the case of MNCs, it was demonstrated that the gene *Slc12a1* encoding NKCC2 transporters is upregulated during an acute hypertonic stimulus (hours) as well as after prolonged water deprivation or salt loading (Konopacka et al., 2015b). The idea is that NKCC2, associated with NKCC1 (Haam et al., 2012), impairs GABA_A_ receptor-mediated hyperpolarization by shifting the E_GABA_ in AVP neurons to a more positive value. Therefore, during AVP-GABA_A_ receptor activation, robust membrane depolarization occurs, so an increase in the secretion of neuropeptides is expected. An integrative view assumes that AVP enhances the activity of NKCC2 expressed in the kidneys through V2-type receptors (Caceres et al., 2009). Therefore, during chronic osmotic challenges, AVP might contribute to increased NaCl reuptake, and in the last instance, aggravate the development of pathologies such as hypertension, edema, and others. However, this is only speculation based on limited evidence.

Another interesting gene enriched in AVP neurons and having increased expression in the SON following water deprivation is *Slc2a13,* coding the H (+)-myo-inositol symporter, which is fundamental for osmotic balance in the brain (Schneider, 2015). This gene was previously demonstrated to increase its expression after hypertonic stimulation in *C. elegans*. Additionally, mutant animals for this gene were hypersensitive to osmotic stress, indicating that this transporter is crucial for the osmoprotective response in *C. elegans* (Kage-Nakadai et al., 2011).

Except for NKCC1, NKCC2 and a few other membrane transporters, there is not much data available about the expression of these other transporters mentioned above or their possible role on MNCs’ response to changes in osmolality. Further studies are necessary to discover their possible regulatory function on the MNC stimulus-synthesis-secretion response.

### Somatodendritic Peptide Release and Osmoregulation

Osmotic challenges also trigger other vital functions in the SON, including the somatodendritic release of AVP and OXT. Although it has been known for 3 decades that neuropeptides are released within the SON (Mason et al., 1986), the variety of roles played by them is not entirely understood.

Initially, the intranuclear release of neuropeptides was described as a crucial self-regulatory mechanism of MNCs because the peptide released would act on their receptors to modulate the neuronal activity (Kombian et al., 1997; Gouzènes et al., 1998; Ludwig, 1998). Hypertonic challenge is a well-known condition that stimulates AVP and OXT secretion as a consequence of changes in the electrical activity of MNCs. However, what worked well for peripheral release did not apply to events occurring within the SON. The dynamics of the somatodendritic release of peptides are different from those of peripheral secretion. In this case, the release is delayed relative to peripheral peptide secretion, and it occurs over a much longer time (Ludwig, 1998). This observation indicates that different regulatory mechanisms are recruited for each compartment. Under physiological conditions, the somatodendritic AVP secretion was shown to be dependent on the firing pattern, i.e., it was rarely observed in response to the continuous firing of action potentials but was intensified by clustered firing activity, a response significantly enhanced by the activation of NMDA receptors (Pitra et al., 2019). Additionally, Gillard et al. (2007) reported consistent evidence that during osmotic challenge, NO is also involved in the local AVP release. According to the authors, the NO produced during hyperosmotic stress indirectly sustains AVP release through a paracrine action, facilitating glutamate/aspartate release. The AVP released also acts back through V2 receptors, increasing the intracellular calcium concentration and potentiating the intranuclear AVP release during strong physiological demand.

The neuropeptides within the SON also mediate communication between neurons and vessels to control the blood flow. It is already known that, unlike in other cells, neurons do not have much reserve of energy, so they use the local vasculature to meet their metabolic demand (Attwell and Laughlin, 2001). Consequently, there is a strong relationship between neuronal activity and blood flow in the brain, a phenomenon called neurovascular coupling (Raichle and Mintun, 2006). In cortical areas, for instance, this phenomenon is well understood, and when cortical neurons increase their activity, the cerebral blood flow also does, to supply this region with the proper amounts of oxygen and glucose (Li et al., 2009). Although the SON is extensively vascularized, it was shown that the metabolic supply of MNCs is independent of its local vasculature, suggesting another function of the coupling in this region (Badaut et al., 2001). A contemporary study demonstrated that acute systemic salt-loading induces intranuclear VP release, which acts through a V1a type receptor to modulate the activity of parenchymal SON arterioles. This interaction results in vasoconstriction and reduced oxygen levels, creating a hypoxic microenvironment, which will promote increased excitability of vasopressin neurons. According to the authors, this works as a positive feedback signaling mechanism during osmotic challenge and characterizes it as an inverse neurovascular coupling in this nucleus (Roy et al., 2021).

Concerning the release of the somatodendritic peptide, the studies presented above shed new light on the possible mechanisms that promote the efficient but not exhaustive secretion of peptides during a challenging situation. So far, the take home message is that somatodendritic release of peptides is essential for the self-control mechanism. However, since the amount of neuropeptides released by dendrites is a hundred to thousand-fold greater than the basal concentration in plasma and cerebral spinal fluid, and their half-life is much longer in the brain (up to 20 min) than in the blood (Ludwig and Leng, 2006), an exciting point to consider is non-canonical roles are played by peptides released in the SON, but this is a subject for another review. Somatodendritic AVP and OXT released act pre- and post-synaptically for self-control of MNCs’ excitability. But this is not their only purpose! They are also involved in the morphological plasticity of glial cells, synaptogenesis, and behavioral regulation, including memory, learning, and social recognition (Mens et al., 1983; Theodosis et al., 1986; Argiolas and Gessa, 1991; Engelmann et al., 1994, 1996; Ludwig and Leng, 2006).

## Conclusion

**FIGURE 4:**
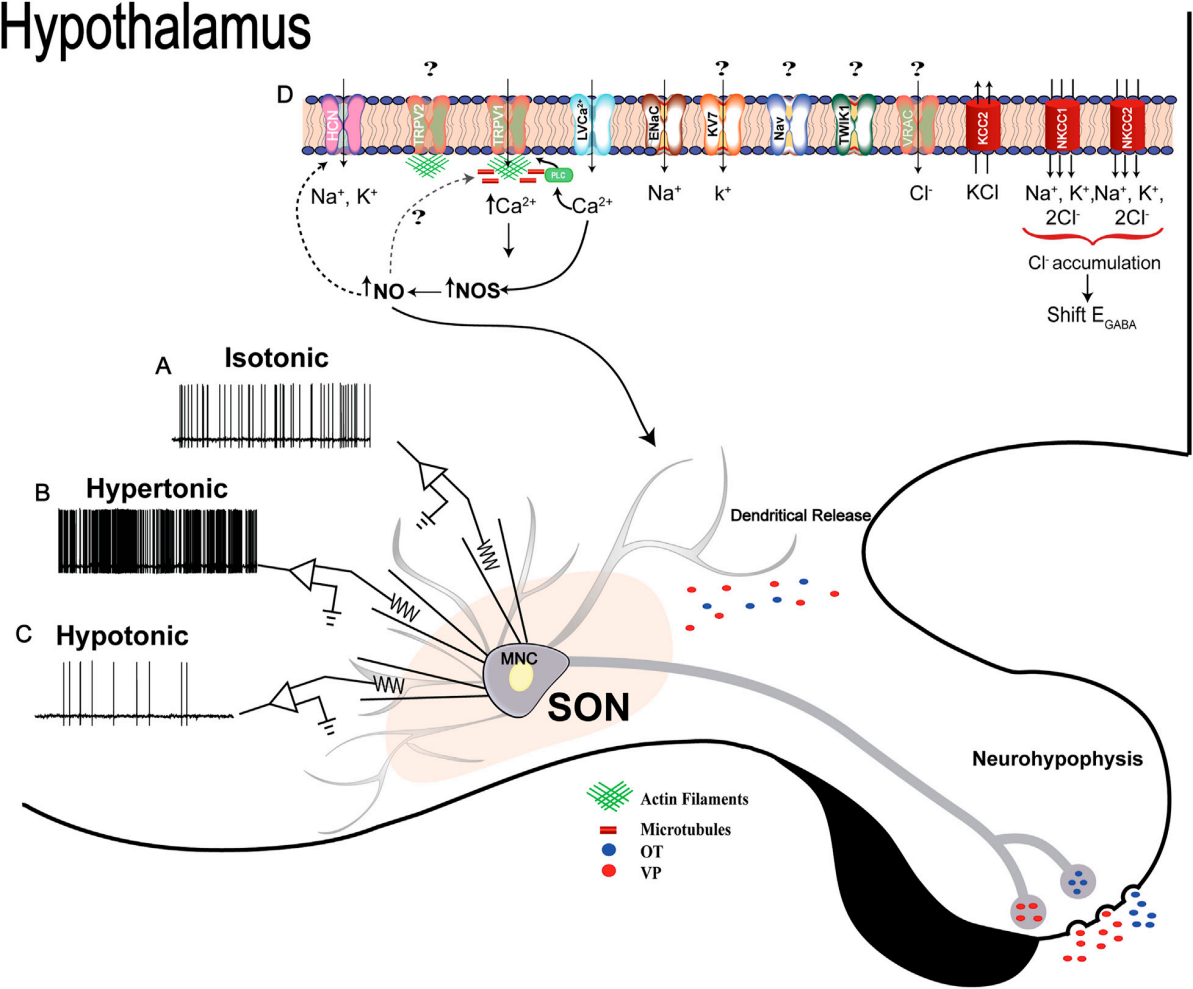
Osmosensitivity of MNCs: **(A, B**, and **C)**, representative traces of intrinsic electrical responses of one MNC in isotonic, hypertonic or hypotonic conditions, respectively, measured using the whole-cell patch-clamp technique. The MNC increases its firing frequency in hypertonic challenges, while the opposite effect is observed during a hypotonic stimulation. The electrical activity of MNCs is directly correlated with neuropeptide secretion, so an increase in AVP and OTX secretion during hypertonicity and a decrease in hypotonicity are expected. Osmotic challenge also triggers AVP and OTX release as a self-regulatory mechanism (details described in the text). **(D)** Schematic representation of ion channels, transporters, and intracellular events recruited after osmotic challenges in MNCs. Although ΔN-TRPV1 has been described as the main molecular mechanism coupling hyperosmolality to electrical responses in the MNCs, Trpv2 was described as the main ion channel gene upregulated in the SON. However, there is no available evidence about the participation of TRPV2 in the MNCs’ osmosensitivity. While microtubules and actin filaments are essential for ΔN-TRPV1 channel activation, TRPV2 seems to interact only with actin filaments. HCN channels, mainly type 3, are also involved in controlling MNCs' electrical properties in hypertonic conditions, and their conductance is modulated by nitric oxide in an S-nitrosylated-dependent mechanism. Sodium non-voltage dependent channels are also part of the mechanisms controlling the excitability of MNCs when the extracellular fluid homeostasis is disturbed by increases in extracellular Na^+^. Potassium voltage-dependent channels (KV7/M-type), in turn, are activated to shape the firing pattern of the neurons during an osmotically evoked activity. Evidence about transporters’ participation in osmoregulation has also emerged. NKCC1, NKCC2 and KCC2 are the principal elements involved. These transporters act in synchrony with several ion channels, particularly chloride (VRAC), to couple changes in osmolality and cell volume of MNCs to the electrical phenomena. Numerous ion channels and transporters also seem to be involved, but further studies are necessary to discover their possible regulatory function in the MNCs stimulus-synthesis-secretion response.

Taken together, the information discussed in this review indicates that several players and mechanisms act in concert to determine the MNCs’ biophysical properties necessary to couple changes in osmolality and cell volume to the electrical phenomena occurring in the plasma membrane (Figure 4). Depending on the timespan of the disturbance, a high degree of morphological and physiological plasticity is needed to sustain the stimulus-activity-secretion response. In other words, unlike short-term stimuli, chronic challenges are multi-dimensional, ranging from gene expression modulation to membrane electrophysiological responses. All changes occur to reconfigure the MNCs’ activity necessary to sustain the release of neuropeptides in response to changing physiological demand. As discussed here, it seems that many questions still require further investigation, by combining state of art techniques ranging from molecules to whole organisms. New functional discoveries related to genes coding membrane channels and transporters that emerged from transcriptome studies in the SON might help in that respect.
